# Further Characterization of the Antiviral Transmembrane Protein MARCH8

**DOI:** 10.3390/cells13080698

**Published:** 2024-04-17

**Authors:** Takuya Tada, Yanzhao Zhang, Dechuan Kong, Michiko Tanaka, Weitong Yao, Masanori Kameoka, Takamasa Ueno, Hideaki Fujita, Kenzo Tokunaga

**Affiliations:** 1 Department of Pathology, National Institute of Infectious Diseases, Tokyo 162-8640, Japan; takuya.tada@nyulangone.org (T.T.); yanzhaozhang@cmu.edu.cn (Y.Z.); dechuan@niid.go.jp (D.K.); yaowt@hbjxlab.com (W.Y.); 2 Department of Microbiology, NYU Grossman School of Medicine, New York, NY 10016, USA; 3 Key Laboratory of Environmental Stress and Chronic Disease Control and Prevention, Ministry of Education, China Medical University, Shenyang 110122, China; 4 Department of Health Laboratory Technology, School of Public Health, China Medical University, Shenyang 110122, China; 5 Joint Research Center for Human Retrovirus Infection, Kumamoto University, Kumamoto 860-8555, Japan; uenotaka@kumamoto-u.ac.jp; 6 Shenzhen Bay Laboratory, Institute of Chemical Biology, Shenzhen 518132, China; 7 Department of Public Health, Kobe University Graduate School of Health Sciences, Kobe 650-0017, Japan; mkameoka@port.kobe-u.ac.jp; 8 Faculty of Pharmaceutical Sciences, Nagasaki International University, Sasebo 859-3298, Japan; fujita@niu.ac.jp

**Keywords:** MARCH8, HIV, VSV-G, species specificity, antiviral activity, cell-to-cell infection

## Abstract

The cellular transmembrane protein MARCH8 impedes the incorporation of various viral envelope glycoproteins, such as the HIV-1 envelope glycoprotein (Env) and vesicular stomatitis virus G-glycoprotein (VSV-G), into virions by downregulating them from the surface of virus-producing cells. This downregulation significantly reduces the efficiency of virus infection. In this study, we aimed to further characterize this host protein by investigating its species specificity and the domains responsible for its antiviral activity, as well as its ability to inhibit cell-to-cell HIV-1 infection. We found that the antiviral function of MARCH8 is well conserved in the rhesus macaque, mouse, and bovine versions. The RING-CH domains of these versions are functionally important for inhibiting HIV-1 Env and VSV-G-pseudovirus infection, whereas tyrosine motifs are crucial for the former only, consistent with findings in human MARCH8. Through analysis of chimeric proteins between MARCH8 and non-antiviral MARCH3, we determined that both the N-terminal and C-terminal cytoplasmic tails, as well as presumably the N-terminal transmembrane domain, of MARCH8 are critical for its antiviral activity. Notably, we found that MARCH8 is unable to block cell-to-cell HIV-1 infection, likely due to its insufficient downregulation of Env. These findings offer further insights into understanding the biology of this antiviral transmembrane protein.

## 1. Introduction

The membrane-associated RING-CH (MARCH) family encompasses 11 members, sharing a common structural framework characterized by two or more transmembrane domains (TMs) and a C4HC3 RING finger (RING-CH) domain located in the N-terminal cytoplasmic tail (CT). This RING-CH domain is pivotal for ubiquitin ligase activity, facilitating interaction with E2 enzymes. Among these family members, MARCH8 is notable for its ability to regulate multiple host membrane proteins, including major histocompatibility complex (MHC)-II [1,2], CD81 [3], CD86 [4], CD44 [4,5], Bap31 [3], TNF-related apoptosis-inducing ligand (TRAIL) receptor 1 [6], CD98 [5], interleukin (IL)-1 receptor accessory protein [7], and transferrin receptor [8].

We previously reported that MARCH8 inhibits virus infection by downregulating HIV-1 envelope glycoprotein (HIV-1 Env) and vesicular stomatitis virus G-glycoprotein (VSV-G) from the surface of virus-producing cells, preventing their incorporation into virions. Unlike VSV-G, which is completely degraded in lysosomes, HIV-1 Env is retained intracellularly without degradation [9]. We later found that VSV-G undergoes lysosomal degradation through MARCH8-mediated ubiquitination at a lysine-rich region (5 out of 29 amino acids) of its CT. In contrast, HIV-1 Env is simply endocytosed in a MARCH8’s tyrosine motif (known as clathrin-dependent endocytic sorting signal)-dependent manner without being degraded. This difference may be attributed to the limited ubiquitination at only two lysine residues out of 151 amino acids in the CT of HIV-1 Env. These observations suggest two different inhibitory pathways mediated by MARCH8: a ubiquitination-dependent pathway for VSV-G and a tyrosine motif-dependent pathway for HIV-1 Env [10,11]. Additionally, our study and others have demonstrated the broad-spectrum inhibition of MARCH8 against various viral envelope glycoproteins [12,13,14,15,16,17,18]. Moreover, our research has elucidated the mechanism by which MARCH8 recognizes the cytoplasmic lysine residues of these glycoproteins, leading to lysosomal degradation [10,11,14,17].

Building upon these findings, our study aimed to further elucidate the antiviral function of MARCH8. We observed that this function, along with the key motifs required for it, is conserved among mammalian MARCH8 proteins, including those from rhesus macaques, mice, and cattle. Crucial determinants were identified in both the N-and C-terminal CTs of MARCH8. However, we noted that MARCH8’s inhibitory function is ineffective against HIV-1 cell-to-cell infection. These results shed light on the intrinsic roles of MARCH8 as an antiviral membrane protein, advancing our understanding of its mechanisms and potential applications in combating viral infections. 

## 2. Materials and Methods

### 2.1. Cells

293T, NIH3T3, and HeLa cells were obtained from the ATCC, whereas MT4 cells were sourced from JCRB Cell Bank. Additionally, RF/6A and MDBK cells were obtained from RIKEN Bio BRC, Kyoto, Japan. MAGIC5 cells were described elsewhere [19]. Either 293T, NIH3T3, HeLa, MDBK, and MAGIC5 cells or MT4 and RF/6A cells were grown in Dulbecco’s Modified Eagle’s Medium (DMEM; GIBCO BRL, Rockville, MD, USA) or RPMI 1640 (GIBCO BRL) supplemented with 10% fetal bovine serum (FBS; Sigma-Aldrich, St. Louis, MO, USA). The cells were tested negative for mycoplasma contamination using the PCR Mycoplasma Detection Set (Takara, Kyoto, Japan).

### 2.2. DNA Constructs

The envelope glycoprotein (Env)-deficient HiBiT-tagged HIV-1 proviral indicator construct pNL-Luc2-IN/HiBiT-E(-)Fin, HIV-1 Env expression plasmid pC-NLenv, VSV-G expression plasmid, pC-VSVg, the human MARCH8 expression plasmid pC-MARCH8, pC-HA-MARCH8, its RING-CH domain mutant pC-MARCH8-W114A, pC-HA-MARCH8-W114A, the tyrosine motif-mutant pC-MARCH8-^222^AxxL^225^, pC-HA-MARCH8-^222^AxxL^225^, the human MARCH3 expression plasmid pC-MARCH3, the lentiviral CRISPR-Cas9 control plasmid pLVTHM, the lentiviral CRISPR-Cas9-MARCH8 knockout plasmid pLV-CRISPR-MARCH8, and the lentiviral packaging vector psPAX2-IN/HiBiT, have previously been described elsewhere [9,15,20,21]. Rhesus macaque, mouse, and bovine versions of MARCH8 expression plasmids, pC-rhMARCH8, pC-muMARCH8, and pC-boMARCH8, were created by inserting KpnI/XhoI fragments of RT-PCR-amplified MARCH8 (from cellular RNAs extracted from RF/6A, NIH3T3, and MDBK cells, respectively) into KpnI/XhoI-digested pCAGGS. The RING-CH mutants of rhesus macaque, mouse, and bovine MARCH8 (pC-rhMARCH8-W114A, pC-muMARCH8-W110A, and pC-boMARCH8-W112A, in which tryptophan residues at positions 114, 110, and 112, respectively, were mutated to alanine residues) were created by inserting overlapping PCR fragments into KpnI/XhoI-digested pCAGGS. Their tyrosine motif mutants (pC-rhMARCH8-^222^AxxL^225^, pC-muMARCH8-^218^AxxL^221^, and pC-boMARCH8-^220^AxxL^223^, in which tyrosine residues at positions 222, 218, and 220 were mutated to an alanine residue) were generated by inserting overlapping PCR fragments into the KpnI/XhoI-digested pCAGGS. The N-terminally HA-tagged versions of all the wild-type and mutant MARCH8 expression plasmids (pC-HA-rhMARCH8, pC-HA-muMARCH8, pC-HA-boMARCH8, pC-HA-rhMARCH8-W114A, pC-HA-muMARCH8-W110A, pC-HA-boMARCH8-W112A, pC-HA-rhMARCH8-^222^AxxL^225^, pC-HA-muMARCH8-^218^AxxL^221^, and pC-HA-boMARCH8-^220^AxxL^223^) were created by inserting PCR-amplified fragments into XhoI/NotI-digested pCAGGS-NHA. The MARCH8/MARCH3-chimeric expression plasmids were created by replacing the N-terminal CT (nCT), N-terminal TM (nTM), ectodomain (EC), C-terminal TM (cTM), or C-terminal CT (cCT) of MARCH8 with PCR-amplified fragments of corresponding MARCH3 domains. Subsequently, their N-terminally HA-tagged versions were created by inserting PCR-amplified fragments into XhoI/NotI-digested pCAGGS-NHA, resulting in plasmids designated as pC-HA-M8/3-nCT, pC-HA-M8/3-nTM, pC-HA-M8/3-EC, pC-HA-M8/3-cTM, and pC-HA-M8/3-cCT. The lentiviral renilla luciferase expression plasmid pWPI-hRL-neoR was generated by inserting the NheI/SpeI fragment of phRL-TK (Promega, E6241) into SpeI-digested pWPI-MCS-neoR. In this construct, a multiple cloning site (BamHI, MluI, SpeI) and the neomycin resistance gene were introduced into the PmeI site and in place of the EGFP gene, respectively, of pWPI. All constructs were verified by the DNA sequencing service FASMAC. Additional information on oligonucleotides can be found in Table S1 in the Supplementary Materials.

### 2.3. Phylogenetic Analysis of the MARCH Proteins

The amino acid sequences of human MARCH family genes and non-human mammalian MARCH8 genes were aligned using the Clustal W (version 1.83). A phylogenetic tree of MARCH family proteins was constructed using the neighbor-joining method, with the reliability of the branching orders determined by the bootstrap approach using MEGA 11.0.13 software [22]. 

### 2.4. Immunoblot Assays

For immunoblot assays, 293T cells were transfected with various MARCH8 expression plasmids using FuGENE6 transfection reagent (Promega, Madison, WI, USA). Following transfection, cells were lysed and subjected to gel electrophoresis. Protein bands were transferred to a nitrocellulose membrane and probed with specific antibodies, including an anti-HA mouse monoclonal antibody (H9658; Sigma-Aldrich) or an anti-β-actin mouse monoclonal antibody (A5316; Sigma-Aldrich). Protein bands were visualized by chemiluminescence using an ECL Western blotting detection system (GE Healthcare, Chicago, IL, USA) and detected with a LAS-3000 imaging system (FujiFilm, Tokyo, Japan).

### 2.5. Production of Pseudotyped Virus and Infectivity Assays

To generate luciferase reporter viruses pseudotyped with either VSV-G or HIV-1-Env, 2.5 × 10^5^ 293T cells were transfected with either increasing amounts (0, 60, or 120 ng) of mammalian MARCH8 expression plasmids (WT, AxxL, and RING-CH mutants) or fixed amounts (100 ng) of MARCH8/MARCH3-chimeric expression plasmids, along with 20 ng of pC-VSVg or pC-NLenv, 500 ng of pNL-Luc2-IN/HiBiT-E(−)Fin, and an empty vector to reach a total DNA amount of 1 μg, using FuGENE6. Sixteen hours post-transfection, the cells were washed with PBS and incubated with 1 mL of fresh complete medium. After an additional 24 h, the supernatants containing the pseudotyped viruses were treated with 37.5 U/ mL of DNaseI (Roche Applied Science, Penzberg, Upper Bavaria, Germany) at 37 °C for 30 min. Viral supernatants were quantified using the HiBiT assay, as described previously [20]. The HiBiT-based luciferase activity in the viral supernatants was measured using a Centro LB960 luminometer (Berthold, Bad Wildbad, Baden-Württemberg, Germany) and converted into p24 antigen levels. To assess viral infectivity, 1 × 10^4^ MAGIC5 cells were exposed to 1 ng of p24 antigen from the HIV-1 supernatants. After 48 h, the cells were lysed in 100 μL of One-Glo luciferase assay reagent (Promega), and firefly luciferase activity was quantified using a Centro LB960 luminometer.

### 2.6. Immunofluorescence Microscopy

HeLa cells were seeded onto collagen-coated 13-mm glass coverslips and transfected with 0.5 μg of plasmids as indicated. After 24 h of culture, the transfected cells were fixed with 4% paraformaldehyde at room temperature for 30 min. The fixed cells were then permeabilized with 0.05% saponin for 10 min. Immunostaining was carried out using 5.0 μg/mL of the anti-HA monoclonal antibody (H9658; Sigma-Aldrich), followed by 5.0 μg/mL of Alexa 488-conjugated goat anti-mouse IgG (A-11001; Molecular Probes, Eugene, OR, USA). All immunofluorescence images were captured using the Fluoview FV1000-IX81 (Olympus, Tokyo, Japan). Alternatively, cells were immunostained with 5.0 μg/mL of the anti-HA monoclonal antibody (H3663; Sigma-Aldrich,), followed by 5.0 μg/mL of Alexa 488-conjugated donkey anti-mouse IgG (A-21202; Molecular Probes,). After immunostaining, F-actin and DNA were stained with 1.0 μg/mL of Phalloidin-TRITC (Sigma-Aldrich, P1951) and 5.0 μg/mL of Hoechst 33342 (B2261; Sigma-Aldrich), respectively. Images were captured using STELLARIS 5 (Leica Microsystems, Wetzlar, Germany).

### 2.7. Primary Cell Culture 

All experiments involving human samples received approval from the Medical Research Ethics Committee of the National Institute of Infectious Diseases, Japan. Peripheral blood mononuclear cells (PBMCs) were obtained from healthy volunteer donors who provided informed consent. PBMCs were isolated using Ficoll-Hypaque gradient centrifugation. Monocytes were subsequently isolated from the PBMCs through positive selection for CD14 using CD14 microbeads (Miltenyi Biotec, Bergisch Gladbach, North Rhine-Westphalia, Germany), then plated at a density of 2.5 × 10^5^ cells per well in 48-well tissue culture plates. The cells were cultured in DMEM supplemented with 10% FBS and 1,000 U/mL macrophage colony-stimulating factor (M-CSF; R&D Systems, Minneapolis, MN, USA) for 10 days to facilitate differentiation into monocyte-derived macrophages (MDMs). The cell culture medium was refreshed every 2 days by replacing half of the cell culture fluid with fresh medium. 

### 2.8. CRISPR-Cas9 Lentiviral Transduction of MDMs

Lentivirus CRISPR stock was generated by cotransfection of 293T cells with pC-VSVg, 0.8 μg of pMDLg/pRRE, 0.2 μg of pCa-Rev, 20 ng of pC-VSVg, and 0.8 μg of the lentiviral CRISPRCas9 expression plasmid (pLV-CRISPR-Ctrl or pLV-CRISPR-MARCH8) with 0.2 μg of an empty plasmid using FuGENE6. After 48 h, the supernatants were treated with DNase I, harvested, and subjected to the HIV-1 p24-antigen capture ELISA (XpressBio, Frederick, MD, USA). MDMs (5 × 10^5^ cells) from two donors were transduced with 1 μg of p24 lentiviruses expressing control CRISPR or CRISPR-targeting MARCH8. The cell lysates from transduced MDMs were analyzed via immunoblotting using the anti-MARCH8 mouse polyclonal antibody (1:1000; A01; Abnova, Taipei, Taiwan) to confirm the effect of MARCH8 knockout.

### 2.9. Cell-Free and Cell-to-Cell Infectivity Assays

For the cell-free infectivity assays, either control or MARCH8-depleted MDMs (2.5 × 10^5^ cells) were infected with 200 ng each of p24 of a VSV-G-pseudotyped HIV-1 luciferase reporter virus that carries an intact Env protein. Sixteen hours later, cells were washed three times with PBS, and 1 mL of complete medium was added. After 24 h, supernatants containing progeny viruses were harvested, subjected to HIV-1 p24-antigen capture ELISA, and the same amounts of viruses were used for infection of MAGIC5 cells. After 48 h, cells were lysed and subjected to the luciferase assay, as described above. For the cell-to-cell infectivity assays, control and MARCH8-depleted MDMs (5 × 10^5^ cells) were similarly infected with 200 ng p24 of the VSV-G-pseudotyped HIV-1 luciferase reporter virus harboring the intact Env protein. Sixteen hours later, the cells were washed with PBS and then incubated with complete medium for 2 days. To prepare target cells, MT4 cells (1 × 10^5^ cells) were transduced with 150 ng p24 of renilla luciferase-expressing lentiviruses, which were produced from 293T cells cotransfected with psPAX2, pWPI-hRL-neoR, and pc-VSVg. After 48 h of transduction, the cells were washed and incubated with 1 mg/mL of G418 (Nacalai Tesque, Kyoto, Japan) for 14 days. Infected MDMs were extensively washed and then cocultured with MT4 cells for 6 h. Cocultured cells were washed with PBS to suspend and collect MT4 cells, which were further cultured in the presence of 160 nM ritonavir (Cayman Chemical, Ann Arbor, MI, USA) for 48 h and subjected to luciferase assays using the Dual-Luciferase Reporter Assay System (E1910; Promega,) with a Centro LB960 luminometer. Infection efficiency in MT4 cells was determined by normalizing firefly luciferase activity to that of renilla.

### 2.10. Statistical Analysis

Column graphs that combine bars and individual data points were created with GraphPad Prism version 10.2. *p* values were generated from two-tailed unpaired *t*-tests for the data.

## 3. Results

### 3.1. Antiviral Function of MARCH8 Is Conserved among Mammals

Previous studies, including our own, have demonstrated the potent antiviral activity of human MARCH8 protein against VSV-G and HIV-1 Env [9,15] as well as a variety of viral envelope glycoproteins [10,11,12,13,14,17]. Notably, Umthong et. al. [17] reported that mouse MARCH8 also targets retroviral envelope glycoproteins. Given these findings, we sought to investigate whether non-human mammalian MARCH8 proteins, including those from rhesus macaque, mouse, and bovine versions, whose sequences are relatively conserved (Figure S1), could similarly inhibit VSV-G- and HIV-1 Env-mediated infection. 

**Figure 1:**
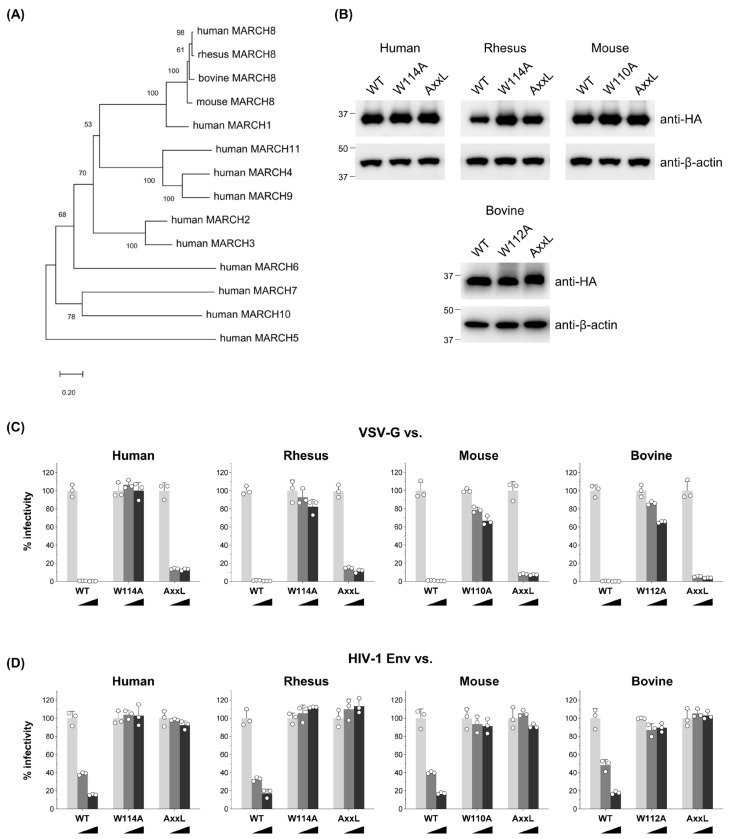
MARCH8′s antiviral function is conserved among mammals. (**A**) An unrooted phylogenetic tree based on MARCH family protein sequences, including mammalian MARCH8 proteins, was generated by the neighbor-joining method. Percent bootstrap values in 1000 replicates are shown on the branches. (**B**) Western blot analysis was performed using lysates from 293T cells transfected with human, rhesus, mouse, or bovine versions of HA-tagged MARCH8 plasmids (wild type (WT), RING-CH domain mutant (W114A, W110A, W112A), or tyrosine motif mutant (AxxL)). Antibodies specific for HA were used to detect MARCH proteins, and β-actin served as an internal control. (**C**,**D**) Viruses were prepared from 293T cells cotransfected with Env-defective HIV-1 luciferase (luc) reporter proviral DNA, either a control or increasing amounts (0, 60, or 120 ng; light grey, dark grey, or black bars, respectively) of human, rhesus, mouse, or bovine MARCH8 plasmids (WT and two mutants), together with VSV-G or HIV-1 Env expression plasmids. Infectivity was determined by infecting MAGIC5 cells with VSV-G-pseudotyped (**C**) or HIV-1 Env-pseudotyped (**D**) luc-reporter viruses and performing luciferase assays. Representative data from three independent experiments are shown as a percentage of the infectivity of control viruses (mean ± S.D., *n* = 3 technical replicates).

We constructed a phylogenetic tree demonstrating that human MARCH families segregate into distinct branches (Figure 1A). Considering that mammalian MARCH8 proteins, including human, rhesus macaque, mouse, and bovine, clustered together, our initial investigation focused on exploring potential similarities in their antiviral activity. To do this, we generated rhesus macaque, mouse, and bovine versions of HA-tagged MARCH8 expression plasmids using cDNA reverse-transcribed from total RNA isolated from RF/6A, NIH3T3, and MDBK cells, respectively. Immunoblotting confirmed MARCH8 protein expression in cells transfected with these plasmids (WT in Figure 1B).

Functional testing of MARCH8 proteins was conducted using viruses produced from 293T cells cotransfected with an Env-defective HIV-1 luciferase reporter proviral DNA and either VSV-G or HIV-1 Env expression plasmids, together with MARCH8 expression plasmids. Similar to human MARCH8, rhesus, mouse, and bovine versions of MARCH8 inhibited both VSV-G- and HIV-1 Env-pseudovirus infection in a dose-dependent manner, demonstrating comparable effectiveness (WT in Figure 1C,D). These findings suggest that the antiviral function of MARCH8 is conserved across mammalian species.

### 3.2. The RING-CH Domain and Tyrosine Motif in Mammalian MARCH8 Are Required for Antiviral Function

The RING-CH domain and canonical tyrosine-based ^222^YxxL^225^ motif, located in the N- and C-terminal CT, respectively, of human MARCH8 protein, have been demonstrated to be essential for its antiviral activity against HIV-1 Env, while only the RING-CH domain is critical for inhibiting VSV-G [9,15,23]. To determine if these sequences are also indispensable in other mammalian MARCH8 proteins, we generated corresponding mutants of MARCH8, confirmed their expression (Figure 1B), and conducted infectivity assays as described above. Our findings revealed that rhesus, mouse, and bovine MARCH8 proteins harboring the W114A mutation exhibited a partial loss of inhibitory activity against both VSV-G- and HIV-1 Env-mediated infection (Figure 1C,D), whereas all the AxxL mutants lost inhibitory activity against only HIV-1, consistent with observations from human MARCH8 mutants (Figure 1D). These results strongly suggest that the requirements of the RING-CH domain and tyrosine motif in mammalian MARCH8 for antiviral function are evolutionarily conserved.

### 3.3. The N-Terminal and C-Terminal CTs of the Human MARCH8 Protein Are Responsible for the Antiviral Activity

**Figure 2:**
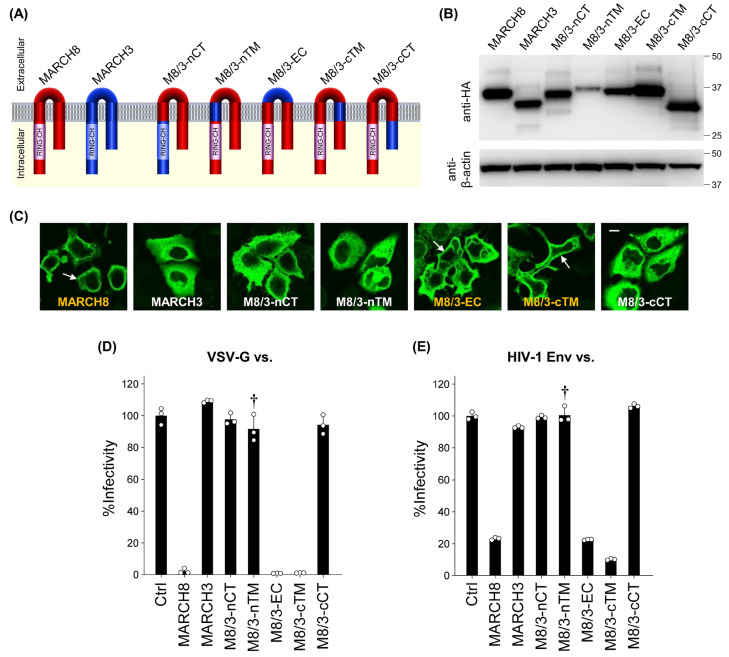
Mapping the domains of the human MARCH8 protein responsible for its antiviral activity. (**A**) The domains of MARCH8 (red) were replaced with those of MARCH3 (blue) as follows: M8/3-nCT: MARCH8 with the N-terminal CT (nCT) of MARCH3. M8/3-nTM: MARCH8 with the N-terminal TM (nTM) of MARCH3. M8/3-EC: MARCH8 with the EC of MARCH3. M8/3-cTM: MARCH8 with the C-terminal TM (cTM) of MARCH3. M8/3-cCT: MARCH8 with the C-terminal CT (cCT) of MARCH3. (**B**) Western blot analysis was performed using lysates from 293T cells transfected with the aforementioned HA-tagged MARCH8/MARCH3 chimera expression plasmids. Antibodies specific for HA were used to detect MARCH proteins, and β-actin was used as an internal control. (**C**) Immunofluorescence microscopy was conducted to analyze the subcellular localization of MARCH8, MARCH3, and their chimeric proteins, using HeLa cells transfected with HA-tagged WT or chimera MARCH expression plasmids. The subcellular localization of chimera MARCH proteins was detected by immunofluorescence microscopy. An anti-HA monoclonal antibody and Alexa 488-conjugated anti-mouse IgG were used as the primary and secondary antibodies, respectively. Arrows indicate plasma membrane localization. Bar, 10 μm. (**D**,**E**) Infectivity assays were performed as described in Figure 1C,D, except that VSV-G-pseudotyped (**D**) or HIV-1 Env-pseudotyped (**E**) viruses were prepared from cells transfected with fixed amounts (100 ng) of MARCH8, MARCH3, and their chimeric plasmids. Representative data from three independent experiments are shown as a percentage of the infectivity of control viruses (mean ± S.D., n = 3 technical replicates). Dagger indicates the presumed loss of antiviral activity despite its Western-blot based lower expression level.

In our previous report, we demonstrated that MARCH3 lacks antiviral activity among different MARCH family proteins that share a similar size and structure (harboring a RING-CH domain and two transmembrane domains (Figure S2)) with MARCH8 [21]. Building upon this observation, we aimed to identify the key domain(s) responsible for the antiviral activity of MARCH8. To achieve this, we constructed five different chimeric plasmids between MARCH8 and MARCH3, termed M8/3-nCT, M8/3-nTM, M8/3-EC, M8/3-cTM, and M8/3-cCT, in which MARCH8 harbors MARCH3’s N-terminal CT, N-terminal TM, EC, C-terminal TM, and C-terminal CT, respectively (Figure 2A). 

Upon transfection of HeLa cells with HA-tagged chimeric MARCH8/3-expressing plasmids, we confirmed equivalent levels of chimeric protein expression in cells transfected with each plasmid (except for M8/3-nTM) through immunoblotting using an anti-hemagglutinin (HA) antibody (Figure 2B). Immunofluorescence microscopy revealed that M8/3-EC and M8/3-cTM were localized to the plasma membrane similar to MARCH8, whereas M8/3-nCT, M8/3-nTM, and M8/3-cCT proteins were diffused throughout the cytoplasmic compartment, as observed in MARCH3. These results imply that the latter three chimeric proteins might have a loss of antiviral activity, resembling MARCH3 (Figure 2C and Figure S3). 

To test this possibility, CD4-positive HeLa-based MAGIC5 cells were infected with viruses produced from cells expressing chimeric MARCH8/3 proteins. Consistent with their subcellular localization, M8/3-nCT, M8/3-nTM, and M8/3-cCT proteins did not inhibit infection by both VSV-G and HIV-1 Env pseudoviruses (Figure 2D,E), whereas M8/3-EC and M8/3-cTM retained their antiviral activity. Although the lower expression level of the M8/3-nTM protein (Figure 2B) prevents a definitive determination of the N-terminal TM as the critical domain, we conclude that both the N-terminal and C-terminal CTs, along with presumably the N-terminal TM domain, are responsible for the antiviral activity in inhibiting HIV-1 and VSV-G pseudovirus infection. 

### 3.4. MARCH8 Is Unable to Block Cell-to-Cell HIV-1 Infection

Given that MARCH8 incompletely inhibited infection by HIV-1 Env-pseudotyped viruses (Figure 1D and Figure 2E) compared with its complete inhibition of VSV-G pseudovirus infection (Figure 1C and Figure 2D; also see refs [9,15]), we hypothesized that this antiviral protein might not be able to impede cell-to-cell HIV-1 transmission, which is known for its higher efficiency than cell-free transmission. To investigate this, we compared the antiviral activity of MARCH8 between cell-free and cell-to-cell HIV-1 infections. 

**Figure 3:**
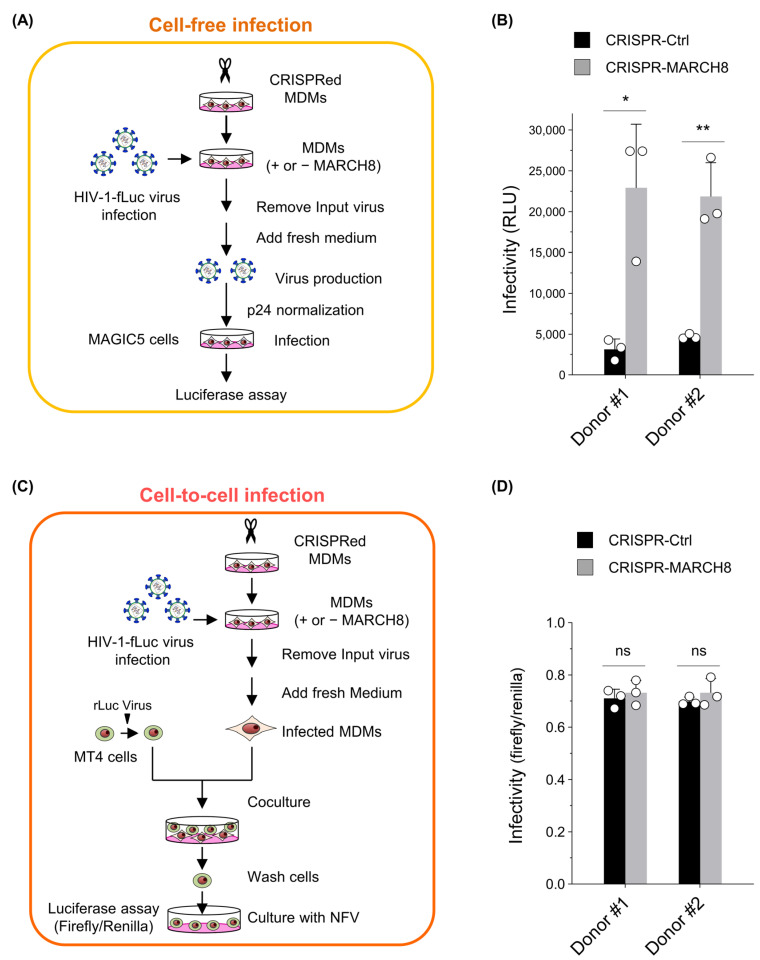
MARCH8 cannot impede cell-to-cell HIV-1 infection from MDMs to CD4-positive T cells. (**A**) Schematic representation of the cell-free infectivity assays. Lentiviral CRISPR-mediated knockout of MARCH8 expression was performed in monocyte-derived macrophages (MDMs) obtained from two donors. Control and MARCH8-depleted MDMs were infected with HIV-1 Env-intact firefly luciferase (fLuc)-reporter viruses, washed, and cultured in fresh media. Infection of MDMs was performed using VSV-G-pseudotyped luc-reporter viruses that carry intact CXCR4-tropic HIV-1 Env, enabling the collection of viruses produced during single-round replication from MDMs without reinfection. Progeny viruses from control and MARCH8-depleted MDMs were normalized for p24 antigen and used to infect MAGIC cells, which were subjected to luc assays. (**B**) Cell-free infectivity of virions produced from control (CRISPR-Ctrl) and MARCH8-depleted (CRISPR-MARCH8) MDMs obtained from two donors (Donor #1 and Donor #2). Data are shown as the fold increase in viral infectivity relative to that of viruses produced from CRISPR-Ctrl MDMs (mean ± S.D. from three independent experiments). * *p* < 0.005, ** *p* < 0.001 compared with the CRISPR-Ctrl using two-tailed unpaired *t*-tests. ns, not significant. (**C**) Schematic representation of the cell-to-cell infectivity assays. Transduction with lentiviral CRISPR and HIV-1 infection were similarly conducted as in (**A**). After infection, MDMs were washed, incubated, and cocultured with MT4 cells that were transduced with renilla luciferase (rLuc)-expressing lentiviruses. MT4 cells were washed, cultured in the presence of nelfinavir (NFV) to block multiple replications, and subjected to dual luc assays. (**D**) Cell-to-cell infectivity in MT4 cells cocultured with CRISPR-Ctrl and CRISPR-MARCH8 MDMs obtained from Donor #1 and Donor #2. Data are shown as the ratio of fLuc/rLuc activity (mean ± S.D. from three independent experiments). The *p* value was calculated using a two-tailed unpaired Student’s *t*-test. ns, not significant.

First, we prepared either control or MARCH8-depleted MDMs by using CRISPR/Cas9 technology (Figure S4) and infected them with the firefly luciferase-reporter HIV-1. For cell-free infection experiments, the progeny viruses produced from infected MDMs, with or without MARCH8 knockout, were normalized and used for cell-free infection of the HeLa-derived CD4-positive cell line MAGIC5 (Figure 3A). Viral infectivity, determined with luciferase assays, was significantly enhanced by MARCH8 depletion in the MDMs derived from two donors (Figure 3B), consistent with our previous findings [9]. 

For cell-to-cell infection experiments, similarly infected MDMs were cocultured with the CD4-positive cell line MT4 transduced with renilla luciferase reporter protein. Subsequently, MT4 cells were separated from MDMs, cultured in the presence of the HIV-1 protease inhibitor nelfinavir, and dual luciferase activities in MT4 cells were measured to determine the efficiency of cell-to-cell infection by normalizing firefly luciferase activity to that of renilla luciferase activity (Figure 3C). Viral infectivity through cell-to-cell infection was almost equivalent in the target MT4 cells that were cocultured with either MARCH8-non-depleted or depleted MDMs derived from two donors (Figure 3D). Hence, we conclude that endogenous levels of MARCH8 expression in MDMs cannot prevent cell-to-cell HIV-1 infection.

## 4. Discussion

In this study, we explored various aspects of MARCH8 biology, shedding light on its antiviral mechanisms: (1) Mammalian MARCH8 proteins exhibit evolutionarily conserved antiviral activity, with key domains and motifs playing crucial roles; (2) Both the N-terminal and C-terminal CTs of MARCH8 are essential for its antiviral function; (3) MARCH8 effectively hinders cell-free HIV-1 infection but fails to impede cell-to-cell transmission. 

From an evolutionary point of view, mammalian MARCH8 proteins tested here, including rhesus macaque, mouse, and bovine versions, were originally expected to harbor an antiviral nature similar to that of the human MARCH8 protein because the sequence of the human version is phylogenetically closer to these mammalian MARCH8 sequences than those of human MARCH1 and MARCH2, which are also antiviral MARCH family members [21,24]. Indeed, as expected, not only all these three MARCH8 proteins inhibited both VSV-G and HIV-1 Env-mediated infection, but also their highly conserved RING-CH domains or tyrosine motifs were found to be antiviral determinants against either both VSV-G and HIV-1 Env or only HIV-1 Env, respectively. Umthong et al. recently showed that mouse MARCH1 was effective against murine leukemia virus (MLV) Env but not HIV-1 Env, whereas mouse MARCH2 had no effect on either MLV or HIV-1 Envs. In contrast, mouse MARCH8 showed antiviral activity against both MLV and HIV-1 Envs [17]. In light of this observation, it would be intriguing to further explore the species specificity of MARCH1 and MARCH2 proteins as well.

In MARCH8/MARCH3 chimeric experiments, we found that both N-terminal and C-terminal CTs of the MARCH8 protein are responsible for inhibiting VSV-G- and HIV-1 Env-mediated infection. The fact that unlike the MARCH8 protein, MARCH3 does not harbor a tyrosine motif in its C-terminal CT (Figure S2) may explain why the M8/3-cCT chimera harboring MARCH3’s CT could not inhibit HIV-1 Env-mediated infection, but it still does not explain why this chimera also lost the ability to inhibit VSV-G-mediated infection that remains sensitive to the tyrosine motif mutant (Figure 1C). Also, despite the fact that MARCH3 also harbors a well-conserved tryptophan residue in the RING-CH domain of the N-terminal CT (Figure S2), why the replacement of the CTs between MARCH8 and MARCH3 affects antiviral function is currently unknown. The observed functional differences in the chimeras may stem, in part, from variances in their binding abilities to HIV-1 Env and VSV-G; however, our preliminary findings could not confirm this conclusively, mainly due to technical challenges in distinguishing specific from non-specific bands to determine MARCH8’s binding region(s) in our IP/Western assays.

The cell-to-cell transmission of HIV-1 has been widely documented as a highly efficient mode of infection compared with cell-free transmission [25,26,27,28,29,30,31,32,33,34,35]. Our previous research has shown that MARCH8 incompletely downregulates HIV-1 Env, resulting in the partial retention of viral infectivity during cell-free infection ([9,15], Figure 1D and Figure 2E). Considering these factors, it is plausible that cell-to-cell HIV-1 infection might be relatively refractory to MARCH8 inhibition compared with cell-free HIV-1 infection. To test this hypothesis, we investigated whether HIV-1 could efficiently transmit to CD4-positive T-cells from MDMs endogenously expressing MARCH8 through cell-to-cell infection. As expected, whereas MARCH8 exhibited potent antiviral activity in cell-free HIV-1 infection, it failed to inhibit cell-to-cell infection. This may be due to the efficient transmission of HIV-1 via virological synapse or tunneling nanotubes/filopodia (for review, see ref [25]), where the lower amount of Env on the surface of MARCH8-expressing cells might still support sufficient HIV-1 transfer. Conversely, MARCH8 may exhibit potent cell-to-cell antiviral activity against certain viruses, such as VSV, lymphocytic choriomeningitis virus, and Chikungunya virus, whose viral membrane proteins are highly susceptible to MARCH8-mediated degradation [9,10,11,15,17,23]. 

In contrast to the antiviral function of MARCH8, which has been described by our current and previous studies [9,10,11,12,13,14,15,17,18], recent research has revealed additional roles for this host factor as a viral cofactor. Kumar et al. demonstrated that MARCH8 supports the assembly of infectious flaviviruses, including hepatitis C virus, dengue virus, and Zika virus [36]. Specifically, in the case of hepatitis C virus, MARCH8 interacts with the cytoplasmic tail of nonstructural protein-2, leading to its polyubiquitination and subsequent recruitment of ESCRT machinery proteins [37], facilitating the intracellular envelopment of the virus [36]. More recently, Khalil et al. identified a crucial role for MARCH8 in stabilizing the papillomavirus oncoprotein E7 in virus-positive cells by degrading CUL1 and UBE2L3, thereby promoting viral infection and persistence [38,39]. Overall, the role of the MARCH8 protein in virus infection appears to be multifaceted, acting as both an antiviral factor and a facilitator of viral processes, like a double-edged sword [11,40].

In conclusion, our study highlights the conservation of MARCH8’s antiviral function across mammalian species, with crucial roles attributed to its RING-CH domain and tyrosine motifs. Furthermore, we have identified the N-terminal and C-terminal CTs of human MARCH8 as crucial components mediating its antiviral activity. However, it is important to note that despite these characteristics, human MARCH8 exhibits limitations in blocking cell-to-cell HIV-1 infection. These findings underscore the complexity of MARCH8-mediated antiviral mechanisms and emphasize the need for additional investigation to elucidate its full potential in combating viral infections. 

## Data Availability

The data presented in this study are available on request from the corresponding author.

## Supplementary Materials

The following supporting information can be downloaded at: https://www.mdpi.com/article/10.3390/cells13080698/s1. Figure S1: Amino acid sequence alignments of the human, rhesus macaque, mouse, and bovine MARCH8 proteins. Amino acid differences are boxed in several colors (polar neutral in green, aliphatic/hydrophobic in orange, aromatic/hydrophobic in purple, basic in red, acidic in blue, unique in pink, and absence in black). EC, extracellular domain; TM, transmembrane domain; CT, cytoplasmic tail. A well-conserved tryptophan residue, which is essential for ubiquitin ligase activity, indicated by a light green arrow; A well-conserved Yxxϕ motif is indicated by a light blue arrow. Figure S2: Amino acid sequence alignments of the human MARCH8 and MARCH3 proteins. Amino acid differences are boxed, as depicted in Figure S1. EC, extracellular domain; TM, transmembrane domain; CT, cytoplasmic tail. A well-conserved tryptophan residue among mammalian MARCH proteins, which is indicated by a light green arrow, is also conserved in the human MARCH3 protein. MARCH8’s Yxxϕ motif in the C-terminal CT, indicated by a light blue arrow, is not seen in MARCH3. Figure S3: Subcellular localization of MARCH8, MARCH3, and their chimeric proteins. HeLa cells transfected with HA-tagged MARCH protein plasmids were analyzed using immunofluorescence microscopy. Anti-HA monoclonal antibody (primary) and Alexa 488-conjugated anti-mouse IgG (secondary) were used for detecting MARCH proteins (green). Hoechst 33342 (blue) and Phalloidin-TRITC (red) were used to stain the nucleus and provide cell morphology, respectively. MARCH8, M8/3-EC, and M8/3-cTM were detected at the plasma membrane (arrows), whereas MARCH3, M8/3-cCT, M8/3-nTM, and M8/3-cTM were exclusively cytoplasmic. Bar, 10 μm. Figure S4: Lentiviral CRISPR-Cas9-mediated knockout (right) of MARCH8 expression in MDMs. Immunoblot images showing the expression of MARCH8 and β-actin in MDMs obtained from two donors. Lentiviral CRISPR-Cas9 technology was employed to knock out MARCH8 expression in MDMs. Table S1: Oligonucleotides used in this study.
