# Supplementary material for: Further Characterization of the Antiviral Transmembrane Protein MARCH8
Source: Cells. 2024 Apr 17;13(8):698. doi: 10.3390/cells13080698 (PMC11049619; doi:10.3390/cells13080698)
Supplement: Supplementary file 1 [file cells-13-00698-s001.zip › cells-2930935-supplementary.pdf]

Supplementary information

Further Characterization of the Antiviral Transmembrane Protein MARCH8

Takuya Tada, Yanzhao Zhang, Dechuan Kong, Michiko Tanaka, Weitong Yao,
Masanori Kameoka, Takamasa Ueno, Hideaki Fujita,
and Kenzo Tokunaga

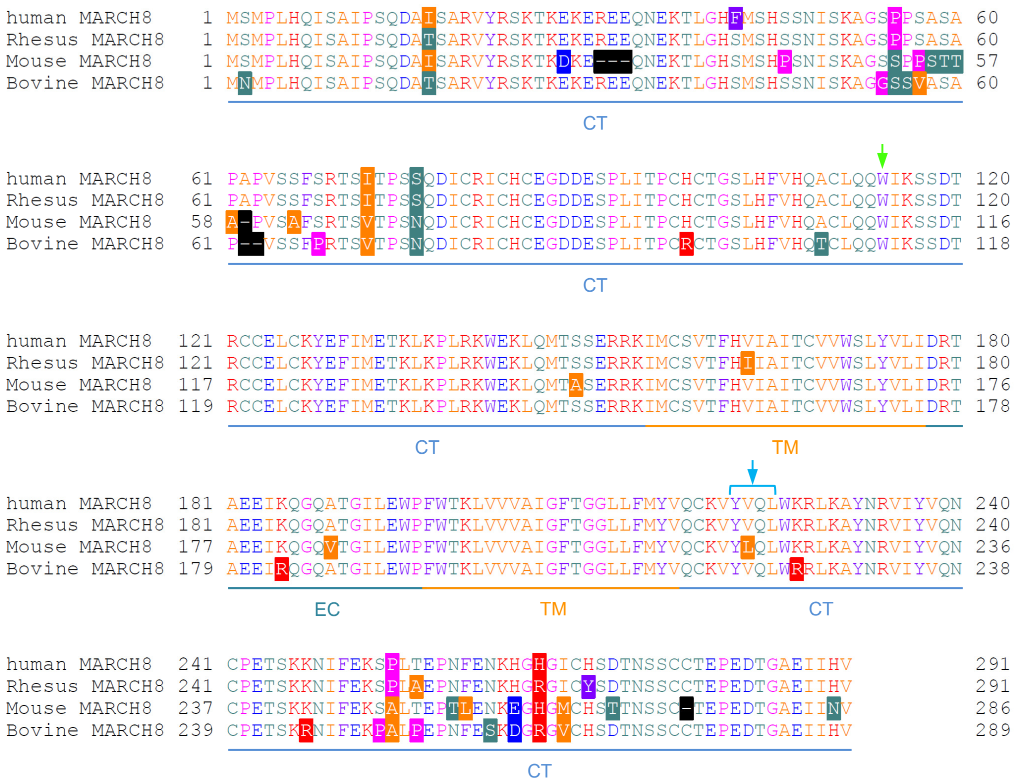

Figure S1. Amino acid sequence alignments of the human, rhesus macaque, mouse, and bovine MARCH8 proteins. Amino acid differences are boxed in several colors (polar neutral in green, aliphatic/hydrophobic in orange, aromatic/hydrophobic in purple, basic in red, acidic in blue, unique in pink, and absence in black). EC, extracellular domain; TM, transmembrane domain; CT, cytoplasmic tail. A well-conserved tryptophan residue, which is essential for ubiquitin ligase activity, indicated by a light green arrow; A well-conserved Yxxϕ motif, indicated by a light blue arrow.

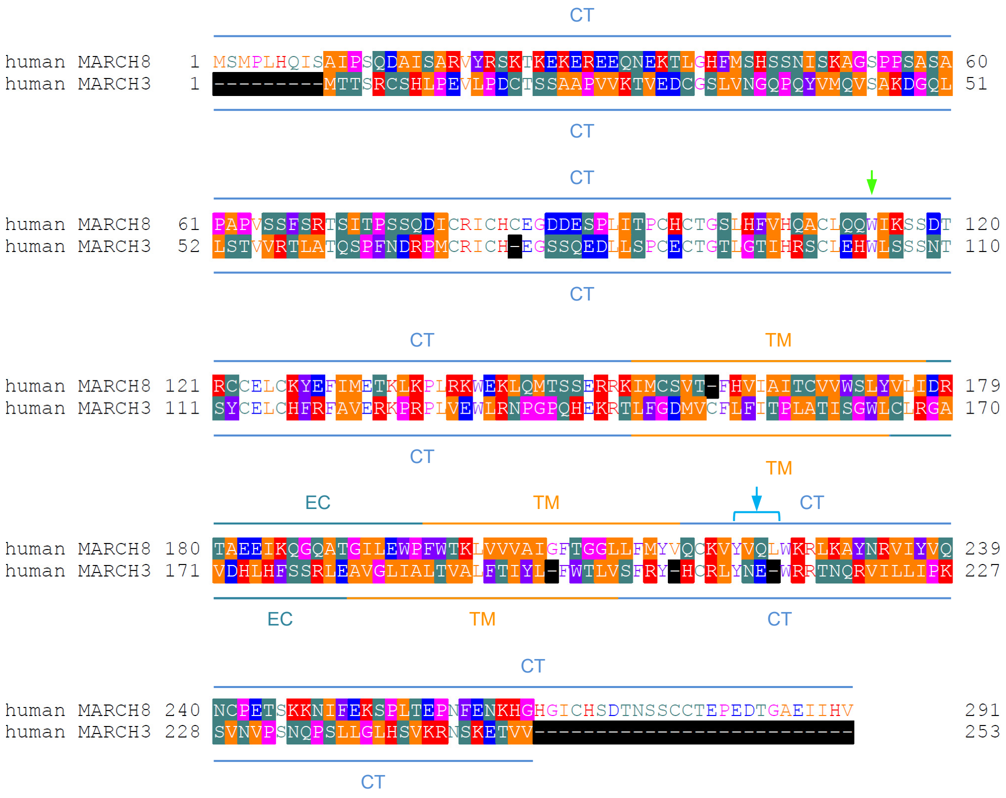

Figure S2. Amino acid sequence alignments of the human MARCH8 and MARCH3 proteins. Amino acid differences are boxed as depicted in Figure S1. EC, extracellular domain; TM, transmembrane domain; CT, cytoplasmic tail. A well-conserved tryptophan residue among mammalian MARCH proteins, which is indicated by a light green arrow, is also conserved in the human MARCH3 protein. MARCH8's Yxxϕ motif in the C-terminal CT, indicated by a light blue arrow, is not seen in MARCH3.

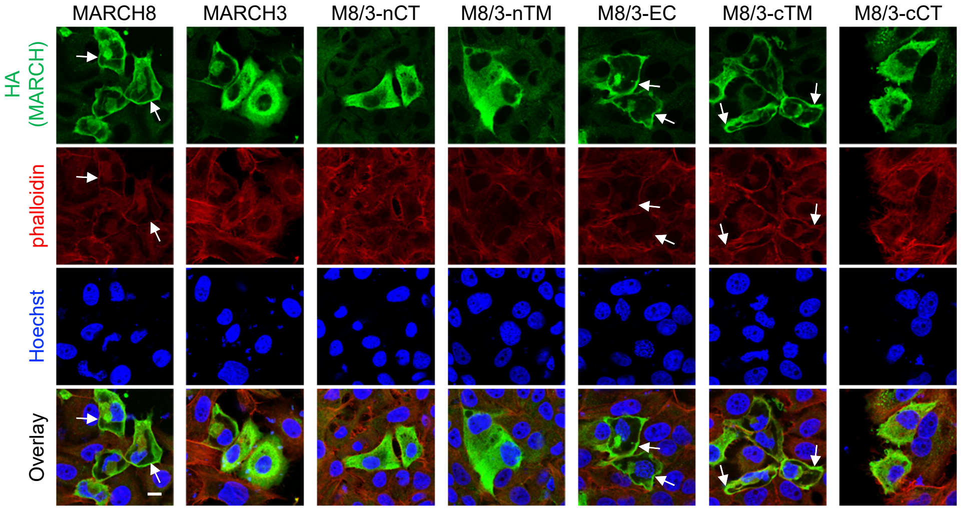

Figure S3. Subcellular localization of MARCH8, MARCH3, and their chimeric proteins. HeLa cells transfected with HA-tagged MARCH protein plasmids were analyzed using immunofluorescence microscopy. Anti-HA monoclonal antibody (primary) and Alexa 488–conjugated anti-mouse IgG (secondary) were used for detecting MARCH proteins (green). Hoechst 33342 (blue) and Phalloidin-TRITC (red) were used to stain the nucleus, and to provide cell morphology, respectively. MARCH8, M8/3-EC, and M8/3-cTM were detected at the plasma membrane (arrows), whereas MARCH3, M8/3-cCT, M8/3-nTM, and M8/3-cTM were exclusively cytoplasmic. Bar, 10 µm.

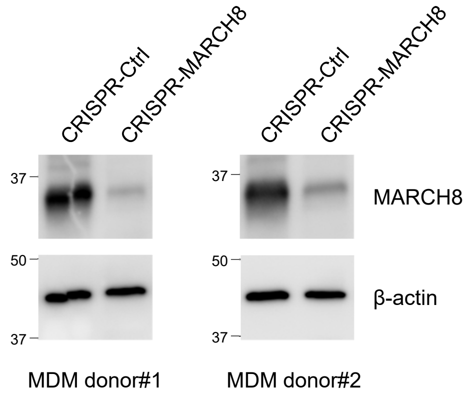

Figure S4. Lentiviral CRISPR-Cas9–mediated knockout (right) of MARCH8 expression in MDMs. Immunoblot images showing the expression of MARCH8 and β-actin in MDMs obtained from two donors. Lentiviral CRISPR-Cas9 technology was employed to knock out MARCH8 expression in MDMs.
